# The Potential Role of Voltage-Dependent Anion Channel in the Treatment of Parkinson's Disease

**DOI:** 10.1155/2022/4665530

**Published:** 2022-10-05

**Authors:** Yajie He, Wenjun Wang, Ting Yang, Elizabeth Rosalind Thomas, Rongyang Dai, Xiang Li

**Affiliations:** ^1^Department of Biochemistry and Molecular Biology, School of Basic Medical Science, Southwest Medical University, Luzhou, China 646000; ^2^Department of Microbiology, North Eastern Indira Gandhi Institute of Health and Medical Science, 793018, Shillong, India

## Abstract

Parkinson's disease (PD) is a neurodegenerative disease second only to Alzheimer's disease in terms of prevalence. Previous studies have indicated that the occurrence and progression of PD are associated with mitochondrial dysfunction. Mitochondrial dysfunction is one of the most important causes for apoptosis of dopaminergic neurons. Therefore, maintaining the stability of mitochondrial functioning is a potential strategy in the treatment of PD. Voltage-dependent anion channel (VDAC) is the main component in the outer mitochondrial membrane, and it participates in a variety of biological processes. In this review, we focus on the potential roles of VDACs in the treatment of PD. We found that VDACs are involved in PD by regulating apoptosis, autophagy, and ferroptosis. VDAC1 oligomerization, VDACs ubiquitination, regulation of mitochondrial permeability transition pore (mPTP) by VDACs, and interaction between VDACs and *α*-synuclein (*α*-syn) are all promising methods for the treatment of PD. We proposed that inhibition of VDAC1 oligomerization and promotion of VDAC1 ubiquitination as an effective approach for the treatment of PD. Previous studies have proven that the expression of VDAC1 has a significant change in PD models. The expression levels of VDAC1 are decreased in the substantia nigra (SN) of patients suffering from PD compared with the control group consisting of normal individuals by using bioinformatics tools. VDAC2 is involved in PD mainly through the regulation of apoptosis. VDAC3 may have a similar function to VDAC1. It can be concluded that the functional roles of VDACs contribute to the therapeutic strategy of PD.

## 1. Introduction

Parkinson's disease (PD) is a progressive neurodegenerative disease, which is very common in the elderly population and sharply increases after the age of 60. According to the study conducted by Global Burden of Disease between 1990 and 2016, PD is the fastest growing neurological disease in terms of death and disability [1]. In most populations, PD is twice as prevalent in men than in women [2, 3]. The clinical manifestations of PD include muscular rigidity, resting tremor, postural instability, dystonia, and dyskinesias [4]. In addition, the non-motor symptoms of PD include depression, anxiety, hallucination, personality changes [5], diarrhea [6], and sleep disorder [7]. Due to the complexity in the diagnosis of PD, PD patients are often dissatisfied with the consultation and treatment process [8]. Based on the clinicopathological studies conducted in Canada and the United Kingdom, the rate of misdiagnosis of PD by clinicians is as high as 25% [9]. The non-motor symptoms of PD are the main reason for high rates of misdiagnosis. Currently, the main drugs used to treat PD are anticholinergic agents, dopamine (DA) receptor agonists, monoamine oxidase B inhibitors, DA replacement drugs, etc. However, all the above-mentioned drugs have side effects such as alimentary distress, insomnia, and dyskinesia [10]. Increasing number of studies have shown that there are significant number of efficient and effective treatment options for PD. The complexity of PD leads to a number of challenges in clinical treatment such as the inability to make an accurate diagnosis at an early stage, lack of specialized clinicians, difficulty to manage symptoms during the later stages of PD, and absence of any medication to stop the progression of PD. Therefore, stopping or delaying the progression of PD is the major challenge to be addressed in the future.

Until now, the cause of PD is not well understood. An increasing body of evidence has suggested that the pathogenesis of PD is related to aging, genetic elements, and environmental factors. It is a well-known fact that with the advancement of age, the chances of suffering from PD increase. According to an epidemiological report by World Health Organization, the prevalence of PD has doubled in the past 25 years. By the year 2019, it is estimated that 8.5 million people worldwide suffer from PD [11]. Among all the neurological disorders, PD is the fastest growing disease and the aging population substantially contributes to this scenario [1]. Vast epidemiological evidences have suggested that aging is associated with PD. Collier et al. have reported that changes in the DA system is correlated to aging [12]. In other words, aging makes dopaminergic neurons more vulnerable to PD. Aging is a factor for triggering PD; however, it may not be the decisive factor. Family history is another risk factor for PD. When compared with the control group, the relative risk for first-degree relatives to suffer from PD increases by about 2 to 3 times [13]. The major pathogenic genes of PD include leucine-rich repeat kinase 2 (LRRK2), *α*-synuclein (SNCA), Parkin RBR E3 ubiquitin protein ligase (PRKN), PTEN-induced putative kinase 1 (PINK1), DJ-1 (Parkinsonism associated deglycase, PARK7), vacuolar protein sorting-35 (VPS35), and Glucosylceramidase (GBA) [14]. Genetic studies confirm that PD is not a disease of a single etiology or natural course. PD is assumed to be caused by mutation in the above-mentioned genes. However, the exact mechanism to trigger mutation remains unclear. Environmental factor is one of the many significant factors that increases the risk of PD. Chen et al. have suggested that in contrast to genetic elements, environmental factors are more modifiable. Therefore, environmental factors are said to possess far-reaching significance in the prevention and treatment of PD [15]. In the past, numerous studies have reported that environmental factors such as traumatic brain injury [16], rotenone [17], paraquat [17], and 1-methyl-4-phenyl-1,2,3,6-tetrahydropyridine (MPTP) [18] can promote the occurrence and development of PD. In addition, caffeine consumption [19], smoking [20], physical activity [21], and use of ibuprofen [22] have a potential role to reduce the risk of PD. Although many studies have indicated that the occurrence of PD is related to various factors, the specific etiology for PD remains unclear.

The main pathological feature of PD is the loss of dopaminergic neurons from the SN of the midbrain, which reduces the DA levels in the brain. Degeneration of the SN will cause the inhibition of the thalamus resulting in a low activation of the motor cortex. Thus, it leads to a decrease in motor activity [23]. During the neurodegenerative process of PD, the aggregation of misfolded *α*-syn forms intracellular inclusions termed Lewy body and Lewy neurite in the neurons, which will result in the degeneration and death of dopaminergic neurons in the striatum. Recent studies have suggested that mitochondrial dysfunction is a major cause for inducing PD [24, 25]. Excessive accumulation of *α*-syn can cause impairment of mitochondrial function in the neurons [26]; however, the mechanism by which *α*-syn disrupts mitochondrial function remains unmapped. Some studies have suggested that the aggregation of *α*-syn can cause decrease in mitochondrial membrane potential and energy production, which induces the release of the pro-apoptotic protein cytochrome c (Cyt c) [27, 28]. Neuronal metabolism requires large amounts of energy from mitochondria; nevertheless, mitochondrial dysfunction often leads to neuronal death. It has been shown that mitochondrial autophagy defect is associated with the pathogenesis of PD [29]. Death of dopaminergic neurons induced by reactive oxygen species (ROS) accumulation has an important role in PD. Mitochondrial dysfunction is closely associated with high levels of ROS production. Mitochondrial respiratory chain complex I is a key site for the production of ROS. Complex I defects are a major cause of apoptosis in neurons and are also thought to be one of the main causes of PD [30]. Thus, the accumulation of ROS is an important pathological feature that induces neuronal damage in PD via mitochondrial-mediated apoptosis. So far, the mechanisms underlying neuronal death in PD remain unmapped. Increasing evidence indicates that mitochondrial dysfunction plays a key role in the pathogenesis of PD [31, 32]. Mitochondria participate in many biological cell processes such as apoptosis [33], autophagy [34], ferroptosis [32], pyroptosis [35], and cellular senescence [36]. Therefore, the improvement of mitochondrial function is an effective method for the treatment of PD.

The voltage-dependent anion channel (VDAC), also called mitochondrial porin, is located in the mitochondrial outer membrane. They belong to the eukaryotic mitochondrial porin family. They function as a gatekeeper during the exchange of molecules and ions between cytosol and mitochondria, thereby controlling the mitochondrial metabolites [37]. Therefore, VDACs can regulate many cellular processes such as calcium homeostasis and oxidative stress. VDACs also have electrophysiological properties and show stability in different membranes [38]. In addition, Rostovtseva et al. have proven that some hydrophobic compounds interacting with the VDACs do not necessarily affect its channel function but can change the physiological functioning of VDACs, thereby affecting the overall functioning of the mitochondria [39]. In mammals, there are three isoforms of VDACs, namely, VDAC1, VDAC2, and VDAC3 [40]. Previous studies on animal models of PD have reported that expression of VDACs has shown significant modification and is closely related to mitochondrial function [41, 42]. VDACs mainly participate in cell energy metabolism by affecting the transport of adenosine triphosphate (ATP) or adenosine diphosphate (ADP) inside and/or outside the mitochondria [43]. Also, VDACs can regulate mitochondrial calcium uptake [44]. Moreover, apart from apoptosis [40] and autophagy [45], recent studies have suggested that VDACs are involved in ferroptosis [46]. Therefore, VDACs exhibit a vulnerability to cell survival. The sensitivity of VDACs to cell survival is due to their involvement in a variety of biological processes such as ATP transportation, energy production, calcium signaling, apoptosis, autophagy, and ferroptosis.

In this review, we focus on the potential role of different isoforms of VDACs during the treatment of PD. This review is expected to enable future researchers to develop groundbreaking treatment that could improve the living condition of PD patients.

## 2. VDAC1 and PD

VDAC1 is a multifunctional protein which is most widely expressed in mammalian mitochondria [47]. Shinohara et al. have proven that the mRNA levels of VDACs found in rat brain can be estimated using Northern blot. Among all the VDACs, VDAC1 is the most expressed protein followed by VDAC2 and VDAC3 [48]. The three-dimensional structure of VDAC1 consists of 19 *β*-strands forming a *β* barrel structure, and N-terminal domain forming an *α*-helical region, which plays a key role in channel function [49, 50]. Knockout mice with the expression of VDAC1 silenced exhibit sensitivity for transport of ADP in oxidative striated muscles, thus affecting the energy metabolism [51]. Deletion of VDAC1 can generate ROS and induce tumor growth in mouse embryonic fibroblasts [52]. The PINK1/Parkin-mediated mitochondrial autophagy pathway has become a promising strategy for the treatment of PD [31]. PINK1 is a serine/threonine kinase. Under mitochondrial stress, PINK1 will promote the recruitment of Parkin to mitochondria, thus inducing autophagy [53]. Parkin functions as an E3 ubiquitin ligase, which can degrade abnormally folded proteins [54]. VDAC1 is one of the key substrates of Parkin regulating autophagy and apoptosis [55]. Lin et al. have mentioned that activating the PINK1/Parkin pathway can promote VDAC1 ubiquitination and mitophagy, thereby protecting neurons from damage [56]. Moreover, Geisler et al. have demonstrated that VDAC1 is required for PINK1/Parkin-mediated autophagy. More importantly, re-transfection of Flag-tagged VDAC1 can rescue Parkin translocation and mitochondrial clearance [57]. Geisler et al. also showed that the autophagy protein p62 can be recruited to the mitochondria by VDAC1, resulting in mitochondrial degradation through lysosomes. Interestingly, Ham et al. have reported that Parkin can ubiquitinate VDAC1 in two different manners including monoubiquitination and polyubiquitination. VDAC1 polyubiquitination is necessary for Parkin-mediated mitophagy in the PD model [55]. The mPTP is a protein found in the inner mitochondrial membrane. mPTP opening is critical for causing cell death. Cui et al. have reported that silencing PINK1 expression in dopaminergic MN9D cells can lead to the activation of mPTP. This indicates that PINK1 is associated with mPTP during the process of PD [58]. A recent study has demonstrated that administration of idebenone can alleviate MPTP-induced PD in mice by regulating VDAC1 expression to activate PINK1/Parkin-mediated mitophagy and reduce dopaminergic neuron loss [59]. In addition, it has been found that PINK1/Parkin-mediated mitophagy is dependent on VDAC1 [57]. Therefore, VDAC1 can probably regulate mitophagy by interacting with PINK1/Parkin pathway in PD. In addition, Chu et al. demonstrated that VDAC1 expression in the SN of patients with PD is significantly decreased compared to the healthy control group [60]. All these studies reveal that VDAC1 is an important target of PINK1/Parkin, which can clearly damage the mitochondria through mitophagy, especially in PD.

**Figure 1:**
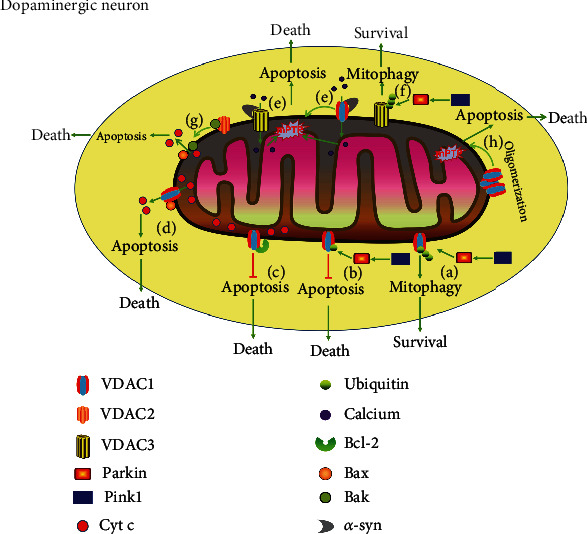
The potential roles of VDACs in PD. (a) VDAC1 polyubiquitination induces mitophagy, thereby promoting the survival of dopaminergic neurons by regulating the PINK1/Parkin pathway in PD. (b) VDAC1 monoubiquitination inhibits apoptosis, thereby promoting the survival of dopaminergic neurons by regulating the PINK1/Parkin pathway in PD. (c) VDAC1 binding to Bcl-2 blocked Cyt c into the cytoplasm and inhibits apoptosis, thereby promoting the survival of dopaminergic neurons in PD. (d) VDAC1 binding to Bax promotes Cyt c into the cytoplasm and activates apoptosis, thereby inducing the death of dopaminergic neurons. (e) VDAC1 or VDAC3 interacting with *α*-syn promotes calcium influx and triggered mPTP, thereby inducing the death of dopaminergic neurons. (f) VDAC3 ubiquitination induces mitophagy, thereby promoting the survival of dopaminergic neurons by regulating the PINK1/Parkin pathway in PD. (g) Dissociation of VDAC2 and Bak induces Cyt c release and promotes apoptosis. (h) VDAC1 oligomerization regulates mPTP to promote apoptosis.

Furthermore, VDAC1 can regulate apoptosis by controlling mPTP [61]. Previous studies have suggested that increased apoptosis often suppresses the induction of autophagy, and the reverse is also true [62, 63]. A recent study has revealed that 4-phenylbutyric acid alleviates rotenone-induced neuronal death in a rat model of PD by targeting and modulating VDAC1-mediated mitochondrial apoptosis [41]. Many studies have reported that *β*-cell lymphoma 2 (Bcl-2) inhibits apoptosis by binding to VDAC1 and blocks apoptotic signals into the cytoplasm such as Cyt c [64–66]. Conversely, pro-apoptotic proteins (such as Bcl-2-associated X protein, Bax) bind to VDAC1 and promote the release of Cyt c [67, 68]. In addition, VDAC1 plays an important role in regulating calcium flux into the mitochondria [69]. Previous studies have suggested that VDAC1 can promote calcium flux into mitochondria followed by mPTP, mitochondrial swelling, and release of Cyt c into the cytoplasm, thus resulting in apoptosis [37, 70–72] (Figure 1). It is worth noting that VDAC1 monoubiquitination has a key role in the pathogenesis of PD by regulating mitochondria-mediated apoptosis [55]. The loss of monoubiquitination of VDAC1 promotes calcium influx through the mitochondrial calcium uniporter (MCU) channel, thereby inducing apoptosis [55]. These results suggest that VDAC1 monoubiquitination can inhibit apoptosis. In addition, erastin, a ferroptosis inducer, induces VDAC1 oligomerization in HT22 cells, although inhibition of VDAC1 oligomerization prevents erastin-induced cell death [73]. More importantly, previous studies have reported that ferroptosis is involved in PD [74]. However, there have been no studies reporting the relationship between VDAC1 and ferroptosis during PD. Therefore, further study is required and VDAC1-ferroptosis relationship could be a potential mechanism for the treatment of PD.

The SH-SY5Y cell line has been widely used to produce impaired DA homeostasis, which is a key aspect in the pathogenesis of PD [75]. Interestingly, Alberio et al. found that administration of DA decreases the expression of VDAC1 in SH-SY5Y cells [76]. Similarly, Premkumar et al. draw the same conclusion. DA administration decreased the expression of VDAC1 in neuronal NMB cells, while overexpression of VDAC1 reduced the neurotoxicity induced by DA [77]. However, the details of this protective mechanism remain unmapped. In addition, there is an increase in the expression of VDAC1 in the PD cell model induced by rotenone [78], 1-methyl-4-phenylpyridinium (MPP^+^) [79], and 6-hydroxydopamine (6-OHDA) [80]. A recent study has revealed that vitamin D has neuroprotective effect on PD model of rats induced by 6-OHDA by decreasing the upregulation of VDAC1 [81]. Increased amount of VDAC1 can probably permit more ubiquitination sites to induce autophagy and inhibit apoptosis, which may be a neuroprotective mechanism. Under oxidative stress, VDAC1 polyubiquitination can induce mitophagy, thereby protecting neurons. VDAC1 monoubiquitination can inhibit apoptosis, thereby protecting neurons (Figure 1). Therefore, it can be suggested that VDAC1 exhibits a promising therapeutic target for PD.

## 3. VDAC2 and PD

VDAC2 is located in the mitochondrial outer membrane and has an important role in the regulation of mitochondria-mediated apoptosis [82]. It is also a mitochondrial membrane porin, allowing diffusion of ions and small hydrophilic molecules of low membrane potential [72, 83]. VDAC2 is involved in a variety of cellular processes distinct from VDAC1, although they share approximately 75% sequence similarity in mammals [84]. Cheng et al. have reported that VDAC2-deficient mouse embryos cannot survive, because of the protective effect against apoptosis. Deletion of VDAC2 makes the cells more susceptible to apoptosis, while re-expression of VDAC2 restores the anti-apoptotic effects [85]. Previous studies have shown that VDAC2 is necessary for the pro-apoptotic activity of Bax in the absence of Bcl-2 homologous antagonist killer (Bak) [86, 87]. VDAC2 exhibits higher calcium permeability compared to VDAC1, even though the quantity of VDAC1 is much more than that of VDAC2 [69]. VDAC2 is not involved in the PINK1/Parkin-mediated mitophagy [88] but is highly involved in apoptosis [87]. VDAC2 can inhibit the mitochondrial apoptotic pathway by interacting with Bak to inhibit its activity [85]. Meanwhile, a previous study has shown that administration of a small molecular compound, WEHI-9625, can stabilize the complex of VDAC2 and Bak thereby inhibiting its dissociation as well as the initiation of apoptosis [89]. Alberio et al. have reported that the expression of VDAC2 is decreased induced by DA in SH-SY5Y cells, which were induced with DA. This was used to reproduce impaired DA homeostasis in PD patients [76]. In addition, it had been shown that erastin binding VDAC2 can change the permeability of the outer mitochondrial membrane, thereby inducing ferroptosis [90, 91]. Many studies have indicated that ferroptosis plays an important role in PD [74]. Therefore, VDAC2 is involved in PD by regulating ferroptosis; however, the specific molecular mechanism still needs to be studied. VDAC2 is not the most abundant isoform; however, many studies have reported that VDAC2 plays an important role in the regulation of apoptosis [87, 92]. Although no specific study has been conducted on the role of VDAC2 in PD, it can be generally stated that VDAC2 plays a key role in various diseases related to the nervous system (Figure 1).

## 4. VDAC3 and PD

VDAC3 is one of the least known isoforms in mammals, which is located in the outer mitochondrial membrane [93]. VDAC3 is considered a mitochondrial sensor for oxidative stress [94]. VDAC3 is transcribed at high levels in the testis of mice [95]. Deletion of VDAC3 causes infertility, while defective VDAC3 causes a significant reduction in sperm motility [96]. Spermatozoa require energy for motility and this energy is obtained from the mitochondria. Reina et al. have proven that VDAC3 is a potential biomarker for mitochondria-related diseases [93]. Interestingly, a recent study has reported that VDAC3 is essential for resistance to ROS-induced oxidative stress [97]. During *in vitro* studies, it has been proven that knockdown of VDAC3 inhibits erastin-induced ferroptosis [98]. There is growing evidence of a relationship between ROS, ferroptosis, and PD. Therefore, it can be suggested that VDAC3 is associated with ROS or/and ferroptosis [99]. So far, the structural framework of VDAC3 has not yet been discovered [100]. Previous studies have demonstrated that the mutation of Parkin gene will trigger dopaminergic neuronal death and thereby induce PD [101, 102]. Mueller et al. have reported that VDAC3 is the main substrate of Parkin in aged Parkin-knockout mice brains [103]. Rosencrans et al. also have proven that VDAC3 plays an important role in PD-associated mitochondrial dysfunction and calcium signaling [69]. VDAC3 can rescue the mitochondrial elimination defect induced by VDAC2 siRNA in VDAC1/3^−/−^ embryonic fibroblasts of mice. In addition, VDAC3 can recruit Parkin and hence depolarize mitochondria, thereby promoting autophagy [88]. Compared to VDAC1, VDAC3 has similar physiological characteristics such as conductance, voltage gating, and anion selectivity. According to the above results, it can be concluded that VDAC3 is mainly involved in autophagy and has potential regulatory effects on PD (Figure 1). At present, there are no reports on the role of VDAC3 during apoptosis [104]. Information regarding VDAC3 functions is still limited. Nonetheless, we believe that VDAC3 is an interesting candidate for PD treatment.

## 5. VDACs and *α*-Syn

A mounting body of evidence has proven that *α*-syn aggregation is associated with the pathogenesis of PD [105]. Electrophysiological experiments have revealed that monomeric *α*-syn can transiently get blocked or be transported through all VDACs isoforms [106]. Risiglione et al. proved that interfering with the interaction between VDACs and *α*-syn may be a very promising strategy for the treatment of PD [104]. Therefore, therapies targeting *α*-syn will play a key role in the treatment of PD. Previous studies have indicated that *α*-syn can reach the inner mitochondrial membrane by interacting with VDAC1 [107, 108]. Overexpression of *α*-syn in the SN of rats by using a recombinant adeno-associated viral vector can lead to the degeneration of dopaminergic neurons by triggering an interaction between *α*-syn and VDAC1, which finally causes changes in the mPTP, thereby resulting in cell death [109]. Chu et al. observed that overexpression of *α*-syn in the SN of rats reduces VDAC1 expression, resulting in mitochondrial dysfunction and cell death [60]. Rosencrans et al. demonstrated that when *α*-syn is added to the reconstituted VDACs, it partially blocks the VDACs' conductance, and VDAC3 exhibits the highest calcium permeability among all the other VDACs [69]. Queralt-Martín et al. showed that VDAC3 cysteine-less mutant has no effect on channel properties; however, it will alter *α*-syn binding kinetics [110]. These results emphasize that the reduced levels of VDACs triggered by *α*-syn accumulation disrupt calcium homeostasis and energy metabolism, resulting in mitochondrial dysfunctions and cell death. Shen et al. suggested that the change of mitochondrial membrane permeability induced by *α*-syn is another important cause for PD [111]. According to the above results, it can be concluded that the downregulated VDACs expression induced by the accumulation of *α*-syn, which reduces the polyubiquitination of VDACs, results in the inhibition of mitophagy. It eventually leads to the death of dopaminergic neurons. Compared to VDAC2, VDAC1 and VDAC3 may play a more important role in PD by interacting with *α*-syn (Figure 1).

## 6. VDACs and Mitochondrial Permeability Transition Pore in PD

VDACs are located in the outer mitochondrial membrane. It is considered to be a key component of the mPTP, although VDACs being a part of mPTP is still controversial [112]. Previous studies have reported that overexpression of VDAC1 can induce apoptosis [113, 114]. It has also been proven that VDACs can exist in different oligomeric states [115]. Increasing evidence has indicated that VDAC1 oligomerization can induce apoptosis by forming mPTP and releasing Cyt c [116, 117]. It has also been reported that the N-terminal *α*-helical domain of VDAC1 is essential for stabilizing the original opening state of VDAC1 [118–120]. In addition, Abu-Hamad et al. reported that the N-terminal region of VDAC1 forms a large enough pore that is critical for the release of Cyt c, which causes apoptosis [121]. VDACs can hetero-oligomerize with the *α*-syn and the complex formed is involved in the misplacement of *α*-syn inside the mitochondria, triggering PD [111, 116]. Hail et al. reported that inhibition of VDAC1 oligomerization can suppress neuronal death and may have protective effects against PD [122]. Based on the above results, it can be concluded that overexpression of VDAC1 can oligomerize VDAC1 to form a flexible pore, thereby controlling Cty c release to induce neuronal death (Figure 1). Most anionic substrates, such as ATP, small cations, and respiratory substrates can enter mitochondria through VDACs. The status of VDACs opening or closing is important for the regulation of mitochondrial function. VDACs in a closed state are prone to trigger apoptosis because it facilitates the entry of Ca^2+^ into the mitochondria [123]. Shen et al. demonstrated that the interaction of the *α*-syn with VDAC1 promotes the opening of the mPTP, and the aberrant mPTP opening will result in the release of Cyt c and mitochondrial swelling [111]. Lu et al. reported that overexpression of *α*-syn in the SN of rats will result in the activation of mPTP by interacting with VDAC1 [109]. In summary, complete shutting or abnormal opening of mPTP induced by VDACs may be an important cause for inducing PD. Therefore, targeting VDACs to regulate mPTP may be a potential approach for the treatment of PD.

## 7. Discussion

PD is the most common neurodegenerative disease second only to Alzheimer's disease in terms of prevalence. So far, the pathogenesis of PD remains unmapped. Current studies have revealed that the occurrence and development of PD are related to aging, genetic elements, and environmental factors. There is no effective drug for the treatment of PD [10]. Although some drugs have been used to treat PD in clinical practice, they are mostly found to have strong side effects. Long-term use of l-3,4-dihydroxyphenylalanine (L-dopa), as DA receptor agonists, induces dyskinesia [124]. Long-term use of amantadine, N-methyl-D-aspartic acid receptor antagonists, causes dizziness, nausea, and insomnia [125]. Administration of pramipexole and ropinirole, DA agonists, induces “sleep attacks” [126]. The existing clinical drugs can only improve or alleviate PD, but cannot cure PD. Studies on PD mice models have revealed that medicinal plants like *Mucuna pruriens* and *Withania somnifera* or herbal compounds like ursolic acid and chlorogenic acid have therapeutic effects from PD and causes minimal side effects [127–130]. It is found that most of these medicinal plants or herbal compounds are able to improve mitochondrial-regulated apoptosis. Improving mitochondrial dysfunction will probably be effective in the treatment of PD and cause minimal side effects. Loss of dopaminergic neurons in the midbrain is found to be one of the most important causes of PD. Mitochondrial dysfunction or injury has always been an important target during the study of PD. Many studies have reported that mitochondrial dysfunction is closely related to apoptosis, autophagy, and ferroptosis [32, 131, 132]. VDACs located in the mitochondrial outer membrane function as a gatekeeper in mediating and regulating a variety of biological processes. Therefore, different isoforms of VDACs play different vital roles in PD. In this review, the potential roles of VDACs are found to be a target in the treatment of PD by regulating calcium homeostasis, apoptosis, and mitophagy. Downregulation of VDAC1 decreases autophagy in a PINK1/Parkin-dependent manner [133]. Upregulation of VDAC1 results in autophagy through the PINK1/Parkin pathway in anoxia/reoxygenation (A/R) model [134]. Many studies have demonstrated that the loss of dopaminergic neurons is mainly due to mitochondrial dysfunction [135, 136]. We speculate that due to the downregulation of VDACs, the level of ubiquitination is decreased, which inhibits autophagy and promotes apoptosis. This results in the loss of dopaminergic neurons. In other words, ubiquitination of VDACs eliminates the damaged mitochondria by regulating mitophagy, thereby inhibiting apoptosis and protecting neurons. Many studies have reported that the autophagy system is impaired in the PD mice model. Postmortem reports of tissue samples collected from PD patients have also reported an impaired autophagy system [137]. In addition, we have also reviewed that VDAC1 can regulate apoptosis. Many researchers have suggested that VDACs binding to Bax/Bcl-2 regulate apoptosis by controlling the release of Cyt c [104]. Based on the above findings, it can be concluded that VDAC1 is a key regulator of PD. It also can be indicated that the ubiquitinated form of VDACs may be a potential target for the treatment of PD. For example, promoting ubiquitination of VDAC1 may be an effective method for the treatment of PD.

Narendra et al. have reported that VDAC2 cannot be ubiquitinated in cells overexpressing Parkin following depolarization [138]. In other words, VDAC2 is probably not involved in the regulation of autophagy. VDAC2 is mainly related to apoptosis. Chin et al. have reported that VDAC2 is necessary for the recruitment of Bax [85, 87]. Although there is no specific study on VDAC2 in PD, we assumed that VDAC2 is involved in the different stages of PD. In the early stage of PD, damaged mitochondria are eliminated through mitophagy, thereby protecting neurons. However, in the advanced stage of PD, damaged mitochondria induces apoptosis, which results in cell death. Therefore, VDAC2 is likely to be involved in the advanced stage of PD.

VDAC3 is also a substrate of Parkin. No reports have indicated any findings regarding the potential role of VDAC3 in apoptosis [104]. A previous study has reported that genes, which are downregulated in PD patients, are enriched in the PD pathway including VDAC3 [139]. Although VDAC3 is the least abundant among the other VDACs, it still plays an important role in PD-associated mitochondrial dysfunction, calcium signaling, and mitophagy [69, 88]. Calcium overload can trigger mPTP, which results in apoptosis [140]. The accumulation and aggregation of *α*-syn triggered by impaired degradation will cause PD [141]. Interestingly, highly soluble *α*-syn causes neurotoxicity of the cell culture model of PD [142]. Previous studies have shown that VDAC3 can interact with *α*-syn [69, 110] to probably induce calcium overload and mitochondrial dysfunction, which may be a potential mechanism of PD (Figure 1).

**Figure 2:**
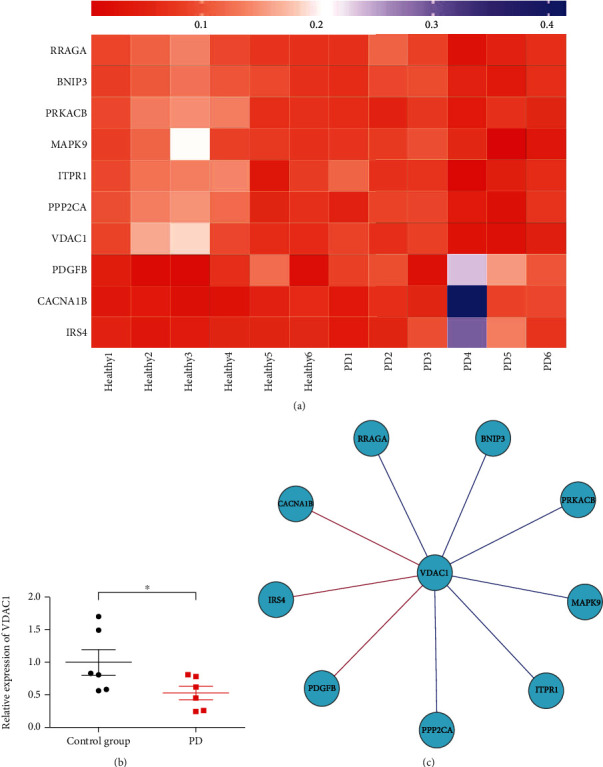
Bioinformatics analysis for VDAC1 and its related genes expression. (a) Heatmap exhibited the expression of VDAC1 and its related genes. (b) The relative expression of VDAC1 in the control group and PD patients. Wilcoxon's test was performed to analyze the VDAC1 expression between control group and PD patients (∗*P* < 0.05, *n* =6). VDAC1 expression was significantly decreased in PD patients compared to control group. (c) VDAC1 correlated with genes in the calcium signaling pathway, apoptosis, and autophagy. (∗*P* < 0.05, *n* =6). Spearman's correlation between VDAC1 and all other genes was computed by R software (version 3.6.3). Significant correlations were chosen with *P* < 0.05 (FDR correction). Red lines linked genes that have negative correlations with VDAC1, and blue lines linked genes that have positive correlation with VDCA1. The positive correlation suggested that two genes have the same varying expression tendency. The negative correlation suggested that two genes have different varying expression tendency.

Further, to explain the significant role of VDACs in PD, we used transcriptome datasets downloaded from NCBI (GSE20333). Heatmap exhibited the expression of VDAC1 and its related genes (Figure 2(a)). We found that expression of VDAC1 in the SN is significantly low in patients with PD when compared to the healthy control group (Figure 2(b)). It can be concluded that the decreased expression of VDAC1 will lead to decreased ubiquitination, which promotes apoptosis and inhibits autophagy. It eventually leads to the death of dopaminergic neurons. The mRNA level of VDAC2 and VDAC3 showed no significant change. This may be due to the following reasons: 1. Limited amount of VDAC2 and VDAC3. 2. Small sample size. 3. Patients affected with different stages of PD.

In addition, we also calculated Spearman's correlation of any of two genes. The genes with a significant correlation (*P* < 0.05) were found to be in coordinated expression. If two genes resulted in a negative correlation, one gene was concluded to be downregulated, while the other was upregulated. If two genes resulted in a positive correlation, both the genes were concluded to either be downregulated or upregulated.  Figure 2(c) shows that VDAC1 possesses significant Spearman's correlations with genes in the calcium signaling pathway, apoptosis, and autophagy, including Ras-related GTP binding A (RRAGA), Bcl-2 interacting protein 3 (BNIP3), protein kinase cAMP-activated catalytic subunit beta (PRKACB), mitogen-activated protein kinase 9 (MAPK9), inositol 1,4,5-trisphosphate receptor type 1 (ITPR1), protein phosphatase 2 catalytic subunit alpha (PPP2CA), platelet-derived growth factor subunit B (PDGFB), calcium voltage-gated channel subunit alpha1 B (CACNA1B), and insulin receptor substrate 4 (IRS4).

Currently, the treatment of PD is mainly based on DA replacement therapy. There is no effective treatment for PD, which drives us to look for new therapeutic agents or targets. Extensive research data suggest a strong relationship between VDACs and PD. Many studies have confirmed that targeting VDAC1 drugs PD such as olesoxime [143], 4-phenylbutyrate [41], and idebenone [59] can improve mitochondrial function and may help to fundamentally alleviate PD. This may be because whatever induces PD will cause mitochondrial dysfunction. Brain neurons, being the most energy-consuming cells, strongly rely on the mitochondria for their energy supply. Therefore, maintaining the integrity of mitochondrial function is of great value for the study of PD. As mentioned above, VDACs via mitochondria are involved in multiple pathways including apoptosis, autophagy, ferroptosis, and Ca^2+^ signaling. Thus, targeted modulation of VDACs is a promising method in the treatment of PD. Ben-Hail et al. discovered a VDAC1-specific inhibitor, which can regulate VDAC1 oligomerization. In addition, this inhibitor prevents the accumulation of ROS, the elevation of Ca^2+^, and the collapse of mPTP [122]. These results suggest that VDAC1-specific inhibitors are effective in protecting neurons from mitochondrial dysfunction by inhibiting apoptosis in multiple ways. Therefore, targeted modulation of VDACs for the treatment of PD has remarkable prospective application for future research.

## 8. Limitations

Apoptosis and impaired autophagy induced by mitochondrial dysfunction are emerging as potentially major features of PD. A detailed understanding of the specific mechanism regulating apoptosis and autophagy is critical for the development of therapies to repair or restore dopaminergic neurons, and VDACs seem to play an important role in this. It can be suggested that activation of autophagy contributes to the alleviation of PD by regulating VDAC1; however, there is no VDAC1-specific autophagy activator. Studies on PD patients, research by Chu et al., and our current study have shown that VDAC1 expression is downregulated during PD [60], but VDAC1 expression is not downregulated *in vivo* and *in vitro* models of PD [41, 42, 59, 78–81]. We speculate that VDAC1 may be a double-edged sword playing different roles in PD. Reduced VDAC1 expression may be due to lack of ubiquitination sites leading to increased apoptosis and inhibition of autophagy in PD models. Elevated VDAC1 expression may be a protective mechanism to inhibit apoptosis and increase autophagy in PD models, but it is important to note that elevated VDAC1 expression may increase VDAC1 oligomerization to induce apoptosis. Therefore, the specific molecular mechanisms by which VDAC1 regulates PD need to be further investigated.

Furthermore, we believe that the combined use of drugs to inhibit the oligomerization of VDAC1 and promote the ubiquitination of VDAC1 may be a very effective approach to suppress PD. This could drive future researchers to develop related drugs or combination of drugs to achieve this effect. At present, there are not many studies on VDAC2/3 and its relation to PD. VDAC2/3's function during PD should be further investigated. More and more studies have shown that VDACs are involved in ferroptosis; however, there have been no reports on the mechanism of action of VDACs in PD by regulating ferroptosis. This also requires further study.

## 9. Conclusion

In this review, the potential roles of VDACs in the treatment of PD can be summarized. VDAC1 and VDAC3 play important roles in PD by regulating autophagy, calcium homeostasis, and apoptosis, while VDAC2 is involved in the regulation of apoptosis in PD. At present, there is no effective drug to treat PD. Therefore, VDACs may be a promising target to treat PD. The development of drugs targeting VDAC1 can possibly be a new way to treat PD. Currently, VDACs have been proven to participate in ferroptosis [91], and many studies have indicated the key role of ferroptosis in PD [74]. Therefore, it can be concluded that VDACs may be a promising target for the treatment of PD by regulating apoptosis, autophagy, and ferroptosis.
